# Dissecting plasmodesmata molecular composition by mass spectrometry-based proteomics

**DOI:** 10.3389/fpls.2012.00307

**Published:** 2013-01-11

**Authors:** Magali S. Salmon, Emmanuelle M. F. Bayer

**Affiliations:** Laboratory of Membrane Biogenesis, CNRS UMR5200, University of BordeauxBordeaux, France

**Keywords:** plasmodesmata, wall, proteomics, subcellular fractionation, *Arabidopsis* suspension cells

## Abstract

In plants, the intercellular communication through the membranous channels called plasmodesmata (PD; singular plasmodesma) plays pivotal roles in the orchestration of development, defence responses, and viral propagation. PD are dynamic structures embedded in the plant cell wall that are defined by specialized domains of the endoplasmic reticulum (ER) and the plasma membrane (PM). PD structure and unique functions are guaranteed by their particular molecular composition. Yet, up to recent years and despite numerous approaches such as mutant screens, immunolocalization, or screening of random cDNAs, only few PD proteins had been conclusively identified and characterized. A clear breakthrough in the search of PD constituents came from mass-spectrometry-based proteomic approaches coupled with subcellular fractionation strategies. Due to their position, firmly anchored in the extracellular matrix, PD are notoriously difficult to isolate for biochemical analysis. Proteomic-based approaches have therefore first relied on the use of cell wall fractions containing embedded PD then on “free” PD fractions whereby PD membranes were released from the walls by enzymatic degradation. To discriminate between likely contaminants and PD protein candidates, bioinformatics tools have often been used in combination with proteomic approaches. GFP fusion proteins of selected candidates have confirmed the PD association of several protein families. Here we review the accomplishments and limitations of the proteomic-based strategies to unravel the functional and structural complexity of PD. We also discuss the role of the identified PD-associated proteins.

## Introduction

**Figure 1:**
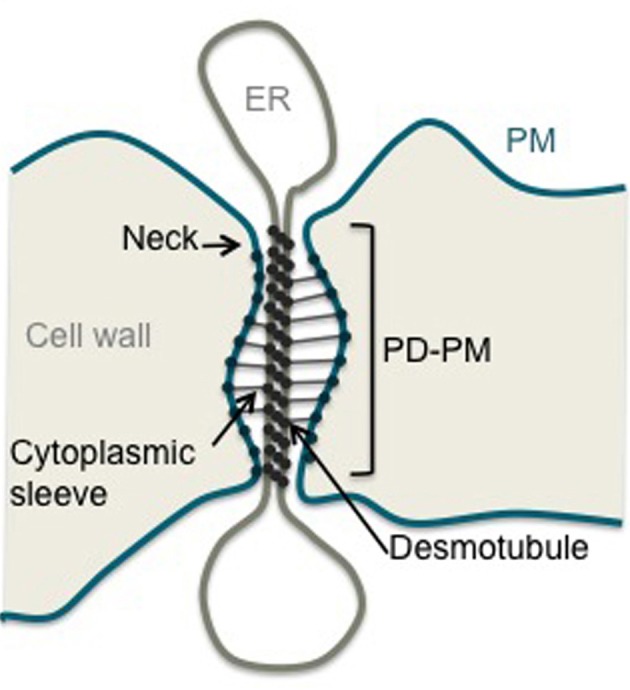
**Structure of a plasmodesma.** Schematic representation of a simple stranded PD. ER, Endoplasmic Reticulum; PM, Plasma Membrane; PD-PM, Plasma Membrane lining PD.

In plants, intercellular communication must overcome the rigid pectocellulosic wall that encompasses all cells. To achieve that plants have developed membranous pores called plasmodesmata (PD) that perforate the extracellular matrix providing symplastic connections between most cell types (Maule, 2008; Xu and Jackson, 2010; Maule et al., 2011). PD are central to a wide range of biological processes that require cell-to-cell communication such as cell fate specification, coordinated growth and development, and transport of carbohydrates. Plant viruses but also fungus can exploit PD transport machinery to establish infection. The emerging view is that PD may well represent a consensus target for pathogens and play a crucial role in defense signaling (Kankanala et al., 2007; Lee and Lu, 2011; Lee et al., 2011). Data regarding PD structure mainly derives from electron microscopy (Helper, 1982; Overall et al., 1982; Tilney et al., 1991; Ding et al., 1992; Botha et al., 1993). PD are lined by the plasma membrane (PM) and contain a central rod, the desmotubule, which is derived from, and continuous with, the endoplasmic reticulum (ER) (Figure 1). Both membrane domains are linked by bridging-like elements whose identity remains a matter of speculation. The space between the PM and the desmotubule is called the cytoplasmic sleeve and provides a conduit through which molecules below the size exclusion limit (SEL) can diffuse between cells in either soluble form or laterally within the membrane phases. Although PD guarantee both cytosolic and membrane continuity between plant cells, the exchange of molecules is under tight control. Non-selective trafficking through diffusion hinges on the number and SEL of PD at a given cellular interface. Both parameters vary depending on the cell type and developmental stage of the tissue considered. An additional level of regulation involves the selective trafficking of specific macromolecules whose size is above the SEL. Such targeted movement implies direct interaction between the trafficking cargo and PD components and results in transient opening of the channels. Understanding of how PD dictate cellular connectivity in such circumstances is dependent on comprehensive knowledge of the composition of PD and functional characterization of their constituents.

## The long quest for plasmodesmal protein constituents

For a long time, the sparse information available about PD constituents has hindered progress in our understanding as to how these membranous structures function. Over the last 30 years the search for PD proteins has been a constant topic of research and endeavors to identify them have employed a wide diversity of approaches (Faulkner and Maule, 2011). Genetic-based approaches have failed to divulge PD structural and regulatory components; this is likely due to the critical role that PD play in growth and development. However, they have supplied critical guidance toward PD functional mechanisms by enabling the identification of proteins, such as a m-type thioredoxin or RNA helicases, which impact on PD permeability but are localized in other subcellular compartments (Kobayashi et al., 2007; Benitez-Alfonso et al., 2009; Stonebloom et al., 2009; Guseman et al., 2010). Targeted approaches aimed at identifying PD receptors have taken advantage of viral movement proteins which accumulate at PD and modify their SEL to permit virus transfer (Benitez-Alfonso et al., 2010). Screens were developed using viral proteins as baits but yielded limited success (Citovsky et al., 1993; Kragler et al., 2000; Paape et al., 2006). Unexpectingly, immunolocalization strategies turned out to be relatively successful. The idea was to identify proteins with established functions that associated with PD. Notably, a close association between PD and elements of the cytoskeleton, especially actin and myosin, were revealed (White et al., 1994; Blackman and Overall, 1998; Radford and White, 1998; Reichelt et al., 1999). They have since been shown to have critical roles in the regulation of cell-to-cell movement and control of PD SEL (White et al., 1994; Ding et al., 1996; Su et al., 2010; White and Barton, 2011; Deeks et al., 2012). Immunological approaches were nevertheless limited to known proteins with available antibodies, and did not lead to unambiguous protein identification.

The need to identify novel PD proteins lead to the development of high throughput screens. Plant cDNAs libraries fused to the fluorescent tag GFP were utilized to this end (Cutler et al., 2000; Escobar et al., 2003). While theoretically appealing, these approaches did not succeed in identifying PD proteins. A different approach for the identification of PD components was required, shifting the focus to the potential for biochemical isolation and proteomic analysis of PD-enriched fractions.

## Purifying PD-enriched subcellular fractions: first steps toward the holy grail

Access to PD structures by subcellular fractionation is rendered difficult both by their location, embedded in the extracellular matrix, and by the small physical contribution they make to total plant tissue mass. In fact, PD are not simply inserted into the wall but firmly anchored into it, probably through the action of proteins and/or wall polymers, that would provide stable bridges between the PM and the wall (Brecknock et al., 2011). Even during an intense plasmolysis treatment, PD stay embedded in the wall matrix while the protoplast retracts (Tilney et al., 1991). However, what was first viewed as a hurdle to PD isolation turned out to be a major advantage. Thus, PD-enriched fractions were readily obtained by purifying wall fragments from plant tissues by mechanical disruption of tissues (French Press, N_2_ pressure bomb, grinding in liquid nitrogen) followed by successive low speed centrifugations to recover and wash wall fragments.

The first attempts to identify PD-associated proteins from purified cell walls, relied on plant tissues known to be rich in PD (Monzer and Kloth, 1991; Kotlizky et al., 1992; Turner et al., 1994; Epel et al., 1995, 1996). With maize mesocotyls as source material, Epel et al. (1996) identified a 41 kDa protein enriched in wall extracts. Screening an expression library, the authors identified Reversibly Glycosylated Polypeptide 2 (RGP2) whose homolog in *Arabidopsis* was subsequently found to be enriched at PD (Sagi et al., 2005). Similarly, monoclonal antibodies raised against maize root tip cell wall proteins (JIM64 and JIM67) were shown to associate with PD in trichomes and mesophyll cells of *N. clevelandii* (Turner et al., 1994; Waigmann et al., 1997) but the identity of their antigen has not yet been retrieved.

**Figure 2:**
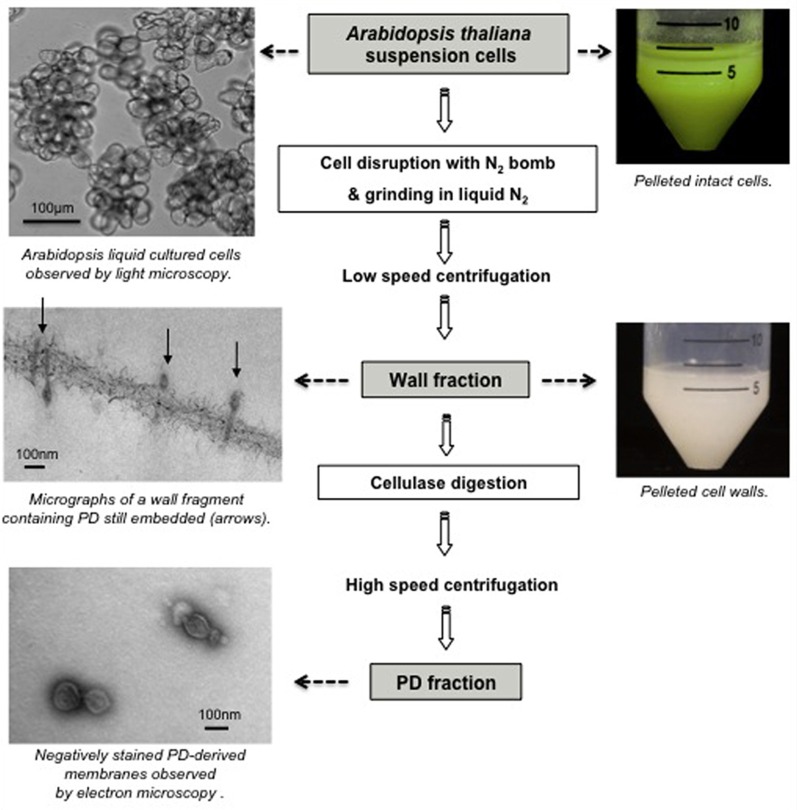
**Purification of PD-enriched wall fraction and “free” PD fraction from *Arabidopsis thaliana* suspension cells**.

Differentiated plant tissues however are often resistant to disruption making the preparation of pure cell wall fractions difficult. This potential drawback is of some importance as the identification of PD components lies in minimizing the level of contamination from intact cells, trapped subcellular organelles, or adhering membranes. As an alternative, the use of liquid cultured cells was investigated by several groups (Lee et al., 2003, 2005; Bayer et al., 2004, 2006; Fernandez-Calvino et al., 2011; Jo et al., 2011). Suspension cells provided an attractive system, as they comprise a friable population of relatively uniform, large cells that lay down abundant primary PD on division walls enabling the recovery of pure wall fractions, containing intact PD (Bayer et al., 2004; Figure 2). Moreover, the amount of plant material that could be processed is not a limiting factor. Using the non-cell-autonomous *Cucurbita maxima* phloem protein (CmPP16) as a bait, the group of Bill Lucas identified a Non-Cell-Autonomous-Protein-Pathway1 (NACPP1; Lee et al., 2003) and recently a Plasmodesmal Germin-like Protein1 (PDGLP1; Ham et al., 2012) from the PD-enriched wall fraction of BY-2 cells. NCAPP1 associates to ER-domains close to the channels where it possibly acts as a shuttle for PD translocation. PDGLP proteins are PD-located and affect root growth when over expressed. Kinase activity essays on the same BY-2 subcellular fraction, lead to the identification of a PD-Associated Protein Kinase (PAPK) that was shown to phosphorylate the movement protein of tobacco mosaic virus (Lee et al., 2005).

With the aim of analyzing the proteome of PD-enriched fraction, Bayer et al. (2004) selected *A. thaliana* suspension culture owing to the extensive genomic information available. Although PD-enriched wall fractions have undoubtedly been of great value in the identification of PD constituents (Lee et al., 2003, 2005; Faulkner et al., 2005; Sagi et al., 2005; Thomas et al., 2008; Simpson et al., 2009; Jo et al., 2011), the contribution of PD proteins to the total wall protein extract was still relatively low. Success in isolating “free” PD from purified cell walls was first reported by Epel group (Epel et al., 1995), with the crucial advance being that PD-derived membranes were released from their position embedded in the wall by treatment with cellulase. This technique was used by Fernandez-Calvino et al. (2011) on *Arabidopsis* cell cultures and produced a final fraction with clear enrichment in known PD-proteins. Ultimately, biochemical fractionation of PD has presented the most straightforward and promising strategy for proteomic-based identification of PD components.

## Combining subcellular fractionation and proteomic approaches to define the PD proteome

Proteomic analyses have emerged as powerful tools for large-scale analysis of complex protein mixtures. Combined with the development of subcellular fractionation strategies these approaches have permitted the identification of an unprecedented number of PD-associated proteins. These technologies have transformed what in the past could only be the result of laborious sequencing of few selected proteins enriched in wall or PD fractions, into a non-targeted approach whereby most, if not all, proteins present in a given sample could be identified.

A limited number of laboratories have actually explored proteomic technologies. Most research teams have only revealed the identity of “confirmed” PD proteins from their proteomic datasets (Sagi et al., 2005; Levy et al., 2007; Jo et al., 2011) but few groups made available the complete list of proteins identified from their PD-enriched fractions (Faulkner et al., 2005; Bayer et al., 2006; Fernandez-Calvino et al., 2011). These publically available databases certainly provide a rich source that can be exploited by all for further identification of PD proteins.

The most comprehensive proteomic analysis of PD proteins was undertaken by the Maule laboratory. Working with *Arabidopsis* suspension cells, the proteome of the wall fraction was first established (Bayer et al., 2006) and with the further refinement in the purification technique, that of the PD fraction (Fernandez-Calvino et al., 2011). Protein MS is coupled and highly dependent on separation strategies that simplify complex biological samples prior to application to the mass analyzer. Sufficient separation is required for both sensitivity and accuracy. Due to the likely hydrophobic nature of PD constituents, gel separation of wall extracts by means of 2D electrophoresis turned out to be inappropriate as most membrane proteins were not resolved (Bayer, unpublished). Instead, a non-gel approach, the Multidimensional Protein Identification Technology (MudPIT; Washburn et al., 2001), which consists of 2D liquid chromatography (2D-LC) directly coupled to a tandem MS, was used to analyze the total wall extract. The subsequent analysis of the PD fraction employed a nano-LC ion trap MS/MS method using an LTQ-Orbitrap™ analyzer that features high resolution, high mass accuracy, and a wide mass-to-charge range (Fernandez-Calvino et al., 2011). Both studies generated exhaustive lists of 792 and 1341 unique protein sequences for the wall and PD fractions, respectively, among which PD components are represented.

## Selecting PD potential candidates from proteomic databases

Sensitive proteomic detection systems have the potential to generate large datasets. Hundreds of proteins can be identified and even with relatively pure samples, minor contaminants are present and cannot be easily discriminated from the proteins of interest. Considering the methodology, what is gained by subcellular fractionation is partially lost by an increase in sensitivity.

To overcome these drawbacks, an elegant approach was developed by the Overall laboratory, who exploited the anatomy of the green alga *Chara corallina* (Blackman and Overall, 1998; Faulkner et al., 2005). The protein profile of wall extracts containing PD (nodal complexes) with those of walls without PD (external internodal walls) were compared by 2D electrophoresis and proteins unique to nodal complexes were analyzed by LC-MS/MS. Some showed sequence similarity to previously identified PD-associated proteins but the approach suffered from the absence of a sequenced genome. A similar approach would be difficult with land plant tissues as virtually all cells are connected with PD.

An alternative strategy consists on downstream analysis of the proteomic datasets generated using bioinformatic tools, databases, and literature sources. This approach was employed by the Maule laboratory following the establishment of *Arabidopsis* cell wall proteome, where PD components accounted for a small proportion of total proteins (Bayer et al., 2006). The selection of potential candidates had to rely on specific characteristics that would distinguish PD-associated proteins from “classical” wall proteins and cytoplasmic contaminants. Since little was known about the structure and function of PD, this was a largely subjective process of elimination. However, based on the nature of PD, the authors argued that a proportion of their protein components would be transported along the secretory pathway to reach either the desmotubule or the PM. Many PD proteins were also expected to be membrane-associated. Candidates were therefore selected based upon two main criteria. First, the preprotein sequence had to contain a N-terminal signal peptide for secretion via the ER and second, to be membrane-associated *via* either a transmembrane domain (TMD) or a Glycosyl Phosphatidyl Inositol (GPI) anchor. A conspicuous drawback of such selection strategy is that it precludes any PD proteins that would associate with PD by other means. A similar strategy was later on also applied to the *Arabidopsis* PD fraction which despite a major enrichment in PD-derived membranes gave rise a colossal proteomic dataset including likely contaminants (Fernandez-Calvino et al., 2011). Jo et al. (2011), who analyzed the wall proteome of rice callus cultures, also focused on membrane-associated proteins to identify PD constituents. The proteomic databases generated from *Arabidopsis* wall and PD fractions were searched using bioinformatic prediction programmes, databases, and published work. In each case about 10% of the proteins identified were shown to fulfill the criteria for PD association and were therefore elected for further analysis. Ultimate confirmation of the physical association of selected candidates with PD structures was then achieved through transient expression of GFP fusion products in leaves and eventually by immunolocalization with electron microscopy. So far, this approach resulted in the conclusive identification of several PD-associated proteins including Plasmodesmata Located Proteins (PDLP; Thomas et al., 2008), Plasmodesmal Callose Binding proteins (PDCB; Simpson et al., 2009), Receptor-Like Kinases (RLK; Fernandez-Calvino et al., 2011), and Tetraspanin (Fernandez-Calvino et al., 2011). We have compiled in Table 1 all PD proteins that have been identified through subcellular fractionation and proteomic-based strategies and confirmed through GFP tagging or immunolocalization.

**Table 1:** **List of confirmed PD-associated proteins identified through subcellular fractionation and proteomic analysis**.

**Protein**	**Gene**	**Biological material used for subcellular fractionation**	**Description and putative function**	**Localization**	**References**
Class1 Reversibly Glycosylated Polypeptide (^C1^RGP)	Ortholog in *Arabidopsis* At5g15650 (AtRGP2)	Maize mesocotyl *N. tabacum* BY-2 suspension cells	May shuttle UDP-sugar to or from glycosyltransferase	PD, Golgi	Epel et al., 1996 Sagi et al., 2005
β1.3 Glucanase	At5g42100	*A. thaliana cry2* mutant	Degradation of callose	PD, PM	Levy et al., 2007
Plasmodesmata Located Protein (PDLP) family	At5g43980	*A. thaliana* suspension cells	Type I membrane receptor Receptor of viral movement protein	PD	Bayer et al., 2006 Thomas et al., 2008
	At3g04370				
	At2g33330				
	A1g04520				
	At3g60720				
	At1g70690				
	At5g37660				
	At2g01660				
Plasmodesmal Callose Binding (PDCB) family	At5g61130	*A. thaliana* suspension cells	Callose binding protein through X8 domain	PD	Bayer et al., 2006 Simpson et al., 2009
	At5g08000				
	At1g18650				
	At1g69295				
	At3g58100				
Leucine Rich Repeat Receptor-Like Kinase (LRR RLK)	At1g56145	*A. thaliana* suspension cells	Signaling	PD, PM	Fernandez-Calvino et al., 2011
*Catharanthus roseus* Receptor-Like Kinase1-like (crRLK1L)	At5g24010	*A. thaliana* suspension cells	Signaling	PD, PM	Fernandez-Calvino et al., 2011
S-domain Receptor-Like Kinase	At4g21380	*A. thaliana* suspension cells	Signaling	PD, PM	Fernandez-Calvino et al., 2011
Tetraspanin3 (TET3)	At3g45600	*A. thaliana* suspension cells	Formation of specialized membrane microdomains	PD, PM	Fernandez-Calvino et al., 2011
Hypothetical protein	At3g15480	*A. thaliana* suspension cells	–	PD, PM	Fernandez-Calvino et al., 2011
Leucine Rich Repeat Receptor-Like Kinase (LRR RLK)	OsO6g47750 OsO2g05960 OsO9g02250	*Rice callus* suspension cells	Signaling	PD	Jo et al., 2011
Lectin Receptor-Like Kinase	Os04g01874	*Rice callus* suspension cells	Signaling	PD	Jo et al., 2011
Wall-Associated Kinase	OsO3g12470 OsO4g51050	*Rice callus* suspension cells	Signaling	PD	Jo et al., 2011
Plasmodesmal-Associated Protein Kinase1 (PAPK1)	Ortholog in *Arabidopsis* At4g28540 (Casein Kinase Like6)	*N. tabacum* BY-2 suspension cells	Signaling Phosphorylation of viral movement protein	PD	Lee et al., 2005
Nt-Plasmodesmal Germin-Like Protein1	Orthologs in *Arabidopsis* At1g09560 (PDGLP1) At1g02335 (PDGLP2)	*N. tabacum* BY-2 suspension cells	Regulation of primary roots growth	PD	Ham et al., 2012

## What have we learnt from proteomic analysis?

These proteomic-based studies, combined with functional analysis of identified PD components, have greatly contributed to elucidate PD organization and regulatory principles. For instance, an interesting finding was that PD house receptor-like activities, such as receptor-like kinases (Fernandez-Calvino et al., 2011; Jo et al., 2011). This implies a role for the channels in signaling events and emphasizes the potential for extracellular stimuli to influence cell-to-cell communication. In the same vein, Thomas et al. (2008) identified from *Arabidopsis* cell wall extracts a new family of receptor-like transmembrane proteins named PDLP which were later on shown to act as receptors for viral movement proteins (Amari et al., 2010). An existing discovery was that PDLP TMD was sufficient for PD targeting indicating that the sorting signals were recognized within the lipid bilayer (Thomas et al., 2008). This, together with the recent finding that lipid rafts, liquid-ordered sterols, and sphingolipids enriched PM microdomains, may associate with PD, raises questions about the role of lipids in defining PD specialized membranes (Raffaele et al., 2009; Mongrand et al., 2010; Tilsner et al., 2011). It is conceivable that the PM region lining PD may itself be sub-divided into functional domains. Sterol-enriched microdomains could well accumulate at the neck region of PD where GPI-anchored proteins such as PDCB or the β1–3 glucanases accumulate to control callose homeostasis and influence PD permeability (Levy et al., 2007; Simpson et al., 2009; Rinne et al., 2011). Hence, GPI anchors preferentially associate with liquid-ordered membrane domains (Sangiorgio et al., 2004; Borner et al., 2005; Kierszniowska et al., 2008). Through its X8 callose-binding domain, PDCB provides a physical link between PD and the wall and may even participate in stabilizing raft domains at PD (Simpson et al., 2009). The presence of functional subdomains at PD is also supported by the presence of TET3 a member of the tetraspanin family (Fernandez-Calvino et al., 2011). Tetraspanins are hydrophobic proteins that have the ability to associate with one another and to recruit specific proteins to build up tetraspanin-enriched microdomains that in mammalian regulate processes such as cell adhesion, signaling, and intracellular trafficking (Stipp et al., 2003; Yunta and Lazo, 2003; Rubinstein, 2011). Like rafts they enable membrane compartmentalization, a process that is required for PD to ensure their unique function.

We must also consider that PD are physically and functionally connected with the endomembrane system. In addition to the continuity of the ER with the desmotubule, the vast majority of PD components identified to date use the secretory pathway for delivery to the channels. For instance, Golgi disrupting treatments prevent both PDLP1 and RGP2 from reaching PD (Sagi et al., 2005; Thomas et al., 2008). Similarly, many plant viruses, which replicate in association with the endomembrane system, traffic to PD along the ER (Niehl and Heinlein, 2011). A number of PD located proteins also associate with the PM (LRR kinases; Jo et al., 2011), the Golgi (RGP2; Sagi et al., 2005), or the ER (calreticulin, Baluška et al., 1999; Chen et al., 2005) highlighting the potential for functional and dynamic relationships with other membrane compartments.

## Conclusion and perspectives

The proteomic-based identification of PD components, combined with imaging techniques, pharmacological, and genetic approaches have brought substantial insight into the complexity of PD structure and dynamics. However, our understanding of PD function is still far from comprehensive and much remains to be determined before we fully comprehend the regulatory mechanisms governing symplastic transport. Many of the identified PD proteins still await functional characterization and advances in this area will provide exciting insights. Moreover, current findings concentrate on proteins with a membrane-localized signature, excluding for instance PD-associated soluble proteins or proteins transiently interacting with the channels which are both likely to be lost during PD purification due to extensive washes with salt containing buffer. Finally, many biological processes governed by symplastic transport probably come with a significant remodeling of PD constituents dictating that there are many more analyses to be done before functional PD components are fully described.

### Conflict of interest statement

The authors declare that the research was conducted in the absence of any commercial or financial relationships that could be construed as a potential conflict of interest.
